# Supplementary material for: A set of multi-entry identification keys to African frugivorous flies (Diptera, Tephritidae)
Source: Zookeys. 2014 Jul 24;(428):97–108. doi: 10.3897/zookeys.428.7366 (PMC4143993; doi:10.3897/zookeys.428.7366)
Supplement: Supplementary material 6 — Key to Ceratitis [file zookeys-428-097-s006.zip › SF6_ZooKeys_key to Ceratitis/key/SF6_ZooKeys_key to Ceratitis/Media/Html/Ceratitis connexa .htm]

Ceratitis (Ceratalaspis) connexa (Bezzi)


***Ceratitis*** **(*Ceratalaspis*)
*connexa* (Bezzi)**

*Hoplolophomyia connexa* Bezzi, 1926: 283.

Body
length: 7.31 (6.85-7.85) mm; wing length: 7.18 (7.00-7.50) mm.

Male

Head.
Antenna yellow to yellow-orange. Third antennal segment twice as long as second
segment. Arista with short hairs over entire length. Frons yellow with lower
half shining silvery; with short scattered hairs which are largely the same
colour as frons; usually convex, sometimes flat, in lateral view slightly
projecting forwards at antennal implant. Lower eye margin without distinct
patch, at most slightly darker yellow colouration. Chaetotaxy normal for
subgenus, except only one frontal bristle developed, sometimes two additional
very small ones.

Thorax.
Postpronotum sligthly paler yellow than mesonotum, almost white; unspotted.
Ground colour of mesonotum pale with yellow tinge; sometimes very pale and
thereby obscuring white patches and lines. Chaetotaxy normal for subgenus.
Scapular setae black. Two anepisternal bristles, rarely three. Scutellum white
basally, otherwise yellow with three black separate markings apically; basally
without dark spots. Subscutellum with three separate spots, same position as
scutellar spots.

Legs
yellow; setation typical for subgenus, mainly pale especially on femora, partly
dark reddish. Posterior and posterodorsal rows on fore femur pale, sometimes
more reddish.

Wing
bands with extensive yellow markings. Banding, marginal band continuous;
cubital band joined with discal band, medial with marginal band; crossvein r-m
at middle of discal cell (occasionally just before middle). Crossvein dm-cu
posteriorly more inwards than anteriorly.

Abdomen.
Yellow ground colour. Anterior margin of fourth abdominal tergite with four and
fifth tergite with two less distinct spots; no distinct banding.

Female

As
male except for the following characters: Antenna yellow. Three frontal
bristles, of approximately equal length. Oviscape shorter than abdominal terga
3-6 combined.

(Description
after De Meyer, 1998)
